# Affective temperaments of Lebanese patients with schizophrenia: comparison by gender and severity of psychosis

**DOI:** 10.1186/s13104-021-05854-8

**Published:** 2021-11-25

**Authors:** Joseph E. Dib, Ilige Nehme, Chadia Haddad, Jocelyne Azar, Souheil Hallit, Sahar Obeid

**Affiliations:** 1 Division of Psychiatry and Clinical Psychology, School of Medicine, University of Nottingham, Nottingham, UK; 2 Faculty of Science, Lebanese University, Fanar, Lebanon; 3 Institute of Epidemiology and Tropical Neurology, INSERM, University of Limoges, CH Esquirol, UMR 1094, Tropical Neuroepidemiology, GEIST, 87000 Limoges, France; 4 Research Department, Psychiatric Hospital of the Cross, Jal Eddib, Lebanon; 5 Faculty of Medicine, Lebanese American University, Byblos, Lebanon; 6 INSPECT-LB: Institut National de Santé Publique, Epidemiologie Clinique Et Toxicologie, Beirut, Lebanon; 7 Faculty of Medicine and Medical Sciences, Holy Spirit University of Kaslik (USEK), Jounieh, Lebanon; 8 Faculty of Arts and Sciences, Holy Spirit University of Kaslik (USEK), Jounieh, Lebanon

**Keywords:** Schizophrenia, TEMPS-A, PANSS, Affective temperaments, Lebanese

## Abstract

**Objectives:**

Our aim in this study was to identify affective temperament differences utilizing the TEMPS-A in a large sample size of Lebanese patients with schizophrenia and compare them to healthy controls. Gender differences, demographic factors and degree of psychotic symptoms were also considered. A cross‐sectional study was conducted at the Psychiatric Hospital of the Cross (PHC) from March to June 2019. Two-hundred fifty chronic patients with schizophrenia were compared to 250 healthy controls randomly chosen from the general population.

**Results:**

Patients with schizophrenia significantly had higher mean depressive, cyclothymic, irritable and anxious temperament scores compared to healthy controls. Healthy controls significantly had a higher mean hyperthymic temperament score compared to patients with schizophrenia. In the group of patients with schizophrenia exclusively, females scored higher in terms of depressive, cyclothymic and anxious temperaments compared to males. In the group of healthy controls, males scored higher in terms of hyperthymic and irritable temperaments compared to females, whereas a higher mean depressive and anxious temperament scores were significantly found in females compared to males. In addition, higher PANSS total scores, as well as higher positive, negative and general subscales scores were significantly associated with higher depressive, cyclothymic, irritable and anxious temperament scores.

**Supplementary Information:**

The online version contains supplementary material available at 10.1186/s13104-021-05854-8.

## Introduction

Individuals with schizophrenia present with varying symptoms and degrees of severity ranging from cognitive, emotional and psychological symptoms that affect their standing in the social and organizational domains [1]. The relationship temperament-psychiatric illness was first recognized by Emil Kraepelin who defined temperaments as personality characteristics whereby abnormal affective states may rise and ultimately lead to a full blown affective illness [2]. Additionally, how personality changes in schizophrenia is still being debated with some citing the personality differences are on a continuum pre and post onset of schizophrenia while others have stated that the personality undergoes a dramatic change only post onset [3, 4]. This, however, has led several researchers to develop temperamental dimensions: irritable, anxious, cyclothymic, depressive and hyperthymic [5, 6]. Irritability is characterized by the tendency to be angry and reactive to slight provocations and disagreements [7]; anxious temperament is characterized by an excessive worry, often leading to impairments in social functioning and other aspects of an individual’s life [8]; cyclothymic temperament is defined as a low grade mood instability often suffering from a recurrence of hypomanic and depressive mood swings [9] and finally, depressive temperament is characterized by low feelings of energy and mood while hyperthymic temperament is characterized by higher levels of energy and elevated mood levels [10].

The study of temperaments in different psychiatric samples have led to the development of the Temperamental Evaluation of Memphis, Pisa, Paris, and San Diego-Auto questionnaire (TEMPS-A) [11]. Temperament refers to innate behavioral style and how this behavior is expressed such as mood, persistence and adaptability; whereas personality is made up of characteristic patterns such as behavior, feelings and thoughts that develops throughout an individuals’ life [12]. This cross-sectional study will focus solely on differences on affective temperaments as it is unable to show the changes of temperaments over time from the onset of schizophrenia. It can, however, highlight the degree of variation between two groups (schizophrenic patients and healthy control group) and might further highlight differences in gender and severity of psychotic symptoms. There have been studies linking temperaments and risk of having a psychotic episode [13–15] specifically how abnormal rates in temperament act as a precursor to a first time onset of schizophrenia [16], which have been conducted in Kenya [15], Finland [14] and Mexico [16].

Gender differences in individuals with schizophrenia are abundant in the psychiatric literature noting variation in symptomology, lifetime risk, course of onset and emotional variability [17]. However, little is known about temperamental differences in gender with patients with schizophrenia, with only one study assessing individuals with schizotypal personality disorder [18]. As for temperamental differences in healthy individuals, there are considerable differences between males and females [19]; while few studies have looked at temperamental differences between patients with schizophrenia and control groups, a multitude has not used the TEMPS-A questionnaire [20, 21] nor have assessed further differences in gender and its relation to severity of psychotic symptoms.

Furthermore, temperaments vary in different cultural settings whether in healthy individuals or within the psychiatric population [22]. As psychiatric research in the Middle East is generally low [23], the objective of this study was to identify affective temperaments in a large sample of Lebanese patients with schizophrenia, compare them to a healthy control group, and then do a comparison by gender, socio-economic factors and severity of psychosis. This study will seek to answer which temperaments would be more present in patients with schizophrenia compared to healthy controls, which temperaments would be more present in females compared to males and which temperaments would be associated with higher levels of psychosis. In this context, we hypothesize that (1) all temperaments would be higher among schizophrenic patients compared to healthy controls; (2) lower hyperthymic, higher depressive, anxious, irritable and cyclothymic temperaments would be associated with more psychosis; and (3) females would present higher levels of cyclothymic, depressive and anxious temperaments and lower irritable and hyperthymic temperaments than males.

## Main text

### Methods

#### Study design and population

A cross‐sectional study was conducted at the Psychiatric Hospital of the Cross (PHC) from March to June 2019, enrolling 306 in-patients with schizophrenia. Patients were randomly chosen according to an online software (www.randomizer.org). Inclusion criteria included patients with schizophrenia (diagnosed by a physician according to the Diagnostic and Statistical Manual of Mental Disorders DSM-5) [24], a duration of hospitalization of more than a year and clinically stable -meaning patients are protected and prevented from harming themselves and/or others. Excluded were patients with schizo-affective disorder, those whose medications doses were variable and those who refused to participate in the study. Two-hundred fifty chronic patients with schizophrenia were compared to 250 healthy controls randomly chosen from the general population. For each patient, a healthy subject from the same gender, region and having the same age was chosen by words of mouth.

#### Data collection and measures

Patients and healthy individuals were interviewed by a clinical neuroscientist. The Arabic questionnaire took 20–30 min to complete. Medical files were assessed for the following information: (i) demographics (age, sex, geographic region, marital status, education level and total monthly salary per household), (ii) clinical information (family history of psychiatric disorders), (iii) social habits (smoking status, alcohol intake).

##### Affective temperament scale (TEMPS‐A)

Validated in Arabic [25], it contains 110 items for females and 109 for males [11] that assess depressive, cyclothymic, hyperthymic, irritable and anxious temperaments (yes/no type of answers). The Cronbach’s alpha coefficients for the depressive temperament was (0.66), cyclothymic (0.83), hyperthymic (0.82), irritable (0.76) and anxious (0.88).

##### Positive and Negative Syndrome Scale (PANSS)

PANSS, validated in Lebanon [26], measures symptom severity of schizophrenia. It evaluates positive, negative and general psychopathology symptoms rated on a 7-point Likert-scale (1  =  absent, 7  =  extreme) [27]*.* Cronbach’s alphas were: positive symptoms (0.877), negative symptoms (0.933) and general psychopathology (0.926).

#### Data analysis

SPSS software v.25 was used for data analysis. Cronbach’s alpha was recorded to ensure the reliability of the scales. The Student’s independent t-test was used to compare continuous variables in two groups, whereas the ANOVA test was used when the comparison involved three or more groups. Pearson correlation was used to correlate two continuous variables. For categorical variables, the Chi-square and Fisher exact tests were used. P  < 0.05 was considered significant.

## Results

### Sample characteristics

Table 1 shows the demographic characteristics of patients with schizophrenia and healthy controls stratified by gender. In the patients with schizophrenia group, males were significantly more likely to be single than females (86.6% vs 69.8%, p  < 0.001), unemployed (73.1% vs 48.3%, p  < 0.001), with a low educational level (less than 8 years; 14.2% vs 3.4%, p  = 0.006) and with a family history of anxiety (98.5% vs 82.8%, p  < 0.001) compared to their female counterparts. In the group of patients with schizophrenia exclusively, a higher mean PANSS negative subscale score was significantly found in females compared to males (13.90 vs. 11.57, p  = 0.031).

**Table 1:** Sociodemographic and clinical characteristics of patients with schizophrenia and healthy control stratified by gender

	Patients with schizophrenia (n = 250)		p value	Healthy controls (n = 250)		p value
	Male	Female		Male	Female	
Education level						
No education	10 (7.5%)	19 (16.4%)	**0.006**	7 (5.2%)	8 (6.9%)	0.889
Less than 8 years	60 (44.8%)	50 (43.1%)		43 (32.1%)	35 (30.2%)	
8 years	45 (33.6%)	43 (37.1%)		54 (40.3%)	44 (37.9%)	
College	19 (14.2%)	4 (3.4%)		30 (22.4%)	29 (25.0%)	
Work status						
Unemployed	98 (73.1%)	56 (48.3%)	**< 0.001**	35 (26.1%)	45 (38.8%)	**0.032**
Employed	36 (26.9%)	60 (51.7%)		99 (73.9%)	71 (61.2%)	
Monthly income						
Less than 1000$	25 (67.6%)	41 (69.5%)	0.447	25 (25.3%)	4 (5.6%)	**0.003**
1000–2000$	12 (32.4%)	18 (30.5%)		62 (62.6%)	53 (74.6%)	
More then 2000$	–	–		12 (12.1%)	14 (19.7%)	
Marital status						
Single	116 (86.6%)	81 (69.8%)	**< 0.001**	44 (32.8%)	21 (18.1%)	**0.002**
Married	7 (5.2%)	21 (18.1%)		69 (51.5%)	77 (66.4%)	
Widowed	0	7 (6.0%)		10 (7.5%)	16 (13.8%)	
Divorced	11 (8.2%)	7 (6.0%)		11 (8.2%)	2 (1.7%)	
Family history of mental illness						
Yes	112 (83.6%)	95 (81.9%)	0.725			
No	22 (16.4%)	21 (18.1%)				
Family history of depression						
Yes	124 (92.5%)	101 (87.1%)	0.151			
No	10 (7.5%)	15 (12.9%)				
Family history of anxiety						
Yes	132 (98.5%)	96 (82.8%)	**< 0.001**			
No	2 (1.5%)	20 (17.2%)				
Family history of suicide						
Yes	126 (94.0%)	106 (91.4%)	0.419			
No	8 (6.0%)	10 (8.6%)				
Family history of drug abuse						
Yes	127 (94.8%)	108 (93.1%)	0.579			
No	7 (5.2%)	8 (6.9%)				

	Mean ± SD	Mean ± SD	p value	Mean ± SD	Mean ± SD	p value
Age	52.34 ± 11.73	50.49 ± 11.19	0.207	51.43 ± 12.13	51.35 ± 10.86	0.961
Duration of illness (in years)	31.85 ± 11.63	30.68 ± 10.75	0.410			
Total PANSS score	50.53 ± 33.87	58.67 ± 38.40	0.076			
Positive subscale	12.13 ± 8.86	13.97 ± 10.54	0.140			
Negative subscale	11.57 ± 8.24	13.90 ± 8.61	**0.031**			
General subscale	26.82 ± 18.86	30.79 ± 21.19	0.119			

Numbers in bold refer to significant p values

*PANSS* Positive and Negative Syndrome Scale

### Affective temperaments differences

Patients with schizophrenia significantly had higher mean depressive, cyclothymic, irritable and anxious temperament scores compared to healthy controls. Healthy controls significantly had a higher mean hyperthymic temperament score compared to patients with schizophrenia (Additional file 1: Table S1).

### Comparison differences by gender

In the group of patients with schizophrenia exclusively, females scored higher in terms of depressive, cyclothymic and anxious temperaments compared to males. In the group of healthy controls, males scored higher in terms of hyperthymic and irritable temperaments compared to females, whereas a higher mean depressive and anxious temperament scores were significantly found in females compared to males (Additional file 1: Table S2).

In addition, higher PANSS total scores, as well as higher positive, negative and general subscales scores were significantly associated with higher depressive, cyclothymic, irritable and anxious temperament scores (Table 2).

**Table 2:** Association between PANSS scores and temperaments in patients with schizophrenia

	Temperaments									
	Depressive	p value	Cyclothymic	p value	Hyperthymic	p value	Irritable	p value	Anxious	p value
	Mean ± SD		Mean ± SD		Mean ± SD		Mean ± SD		Mean ± SD	
PANSS^a^										
Mild	0.62 ± 0.14	**0.005**	0.47 ± 0.17	**< 0.001**	0.50 ± 0.17	0.988	0.37 ± 0.21	0.127	0.49 ± 0.24	**0.015**
Moderate	0.65 ± 0.16		0.53 ± 0.13		0.50 ± 0.15		0.41 ± 0.18		0.59 ± 0.17	
Marked	0.68 ± 0.12		0.58 ± 0.13		0.49 ± 0.16		0.44 ± 0.17		0.57 ± 0.17	
Severe	0.70 ± 0.14		0.58 ± 0.13		0.49 ± 0.19		0.44 ± 0.18		0.59 ± 0.18	

	Correlation coefficient	p value	Correlation coefficient	p value	Correlation coefficient	p value	Correlation coefficient	p value	Correlation coefficient	p value
Total PANSS score	0.257	**< 0.001**	0.340	**< 0.001**	− 0.018	0.776	0.213	**0.001**	0.220	**< 0.001**
Positive subscale	0.178	**0.005**	0.296	**< 0.001**	− 0.026	0.688	0.150	**0.018**	0.151	**0.017**
Negative subscale	0.219	**< 0.001**	0.306	**< 0.001**	− 0.020	0.753	0.192	**0.002**	0.201	**0.001**
General subscale	0.285	**< 0.001**	0.342	**< 0.001**	− 0.012	0.851	0.231	**< 0.001**	0.239	**< 0.001**

Depressive temperament: mild vs. moderate (0.62 vs. 0.65, p  = 1.000); mild vs. marked (0.62 vs. 0.68, p  = 0.064); mild vs. severe (0.62 vs. 0.70, p  = 0.020); moderate vs. marked (0.65 vs. 0.68, p  = 1.000); moderate vs. severe (0.65 vs. 0.70, p  = 0.812); marked vs. severe (0.68 vs. 0.70, p  = 1.000)

Cyclothymic temperament: mild vs. moderate (0.47 vs. 0.53, p  = 0.416); mild vs. marked (0.47 vs. 0.58, p  = 0.001); mild vs. severe (0.47 vs. 0.58, p  = 0.008); moderate vs. marked (0.53 vs. 0.58, p  = 1.000); moderate vs. severe (0.53 vs. 0.58, p  = 1.000); marked vs. severe (0.58 vs. 0.58, p  = 1.000)

Hyperthymic temperament: mild vs. moderate (0.50 vs. 0.50, p  = 1.000); mild vs. marked (0.50 vs. 0.49, p  = 1.000); mild vs. severe (0.50 vs. 0.49, p  = 1.000); moderate vs. marked (0.50 vs. 0.49, p  = 1.000); moderate vs. severe (0.50 vs. 0.49, p  = 1.000); marked vs. severe (0.49 vs. 0.49, p  = 1.000)

Irritable temperament: mild vs. moderate (0.37 vs. 0.41, p  = 1.000); mild vs. marked (0.37 vs. 0.44, p  = 0.376); mild vs. severe (0.37 vs. 0.44, p  = 0.497); moderate vs. marked (0.41 vs. 0.44, p  = 1.000); moderate vs. severe (0.41 vs. 0.44, p  = 1.000); marked vs. severe (0.44 vs. 0.44, p  = 1.000)

Anxious temperament: mild vs. moderate (0.49 vs. 0.59, p  = 0.114); mild vs. marked (0.49 vs. 0.57, p  = 0.200); mild vs. severe (0.49 vs. 0.59, p  = 0.236); moderate vs. marked (0.59 vs. 0.57, p  = 1.000); moderate vs. severe (0.59 vs. 0.59, p  = 1.000); marked vs. severe (0.57 vs. 0.59, p  = 1.000)

Numbers in bold refer to significant p value

*PANSS* Positive and Negative Syndrome Scale

^a^Post hoc analysis for PANSS categories taking the temperaments as the dependent variables

## Discussion

In this study, we aimed to compare affective temperament between patients with schizophrenia and a control group with no history of mental disorders as well as gender differences and degree of psychosis. To our knowledge, this was the first study of its kind in the Middle East.

Within affective temperament comparing both groups, depressive, cyclothymic, irritable and anxious temperaments were significantly higher in patients with schizophrenia when compared to healthy controls with the exception of hyperthymic being lower in the schizophrenia group when compared to the healthy group. As mentioned in the introduction, temperament is innate and the question if temperamental factors increase in the onset of schizophrenia is beyond the design scope of this study. Given the significant differences, it can be assumed a range of both biological and social factors contribute to these differences. As shown in Table 2, significant factors such as history of anxiety in the family as well as higher scores on the PANSS could highlight the differences in temperament.

Within the schizophrenia group, patients from both genders also scored higher in depressive and anxious temperament similar to other studies [28, 29]. Patients with schizophrenia suffer from stigma causing overwhelming stress that impacts them on the cognitive, emotional and social level which attribute to depressive and anxious symptoms [30]. Cyclothymic temperament was higher in individuals with schizophrenia. While the majority of work has compared cyclothymia to bipolar disorder, it has been noted that individuals with schizophrenia are at a higher risk of having cyclothymic symptoms which has also been linked as a predictive temperament of developing the disorder [31].

Assessing gender differences, females in the schizophrenia group scored higher on average when compared to their male counterparts on cyclothymic, depressive and anxious temperament which is congruent with the current literature [32]. Significantly higher depressive and anxious mean were found in females with schizophrenia compared to healthy females. In healthy subjects, men had a significantly higher mean in both hyperthymic and irritable temperaments compared to healthy women which could be attributed to the presence of higher levels of testosterone in males [33]. These gender differences of temperament can be due to hormonal differences between men and women [32].

Results showed that higher total PANSS scores, negative PANSS and general PANSS were significantly associated with higher mean of depressive, irritable and cyclothymic temperament, in concordance with other studies [20, 34]. The presence of positive and negative symptoms severely impacts the dynamics of social interactions making it difficult to establish a positive relationship which can often be frustrating to both the individual with schizophrenia and healthy subjects [35].

Looking at sociodemographic outcomes, people diagnosed with schizophrenia fare poorly compared with those who do not meet the diagnosis, especially at being unemployed and being single [36]. Individuals in the schizophrenia group also had poorer levels of education, which ultimately affects their work status. This in turn affects their employment chances making it difficult to support themselves.

## Conclusion

This is the first study of its kind using the TEMPS in patients with schizophrenia while assessing the comparison between gender and the severity of psychotic symptoms. Both males and females fared poorly in the schizophrenia group compared to healthy controls. The schizophrenia group tended to have higher levels of anxious, cyclothymic, depressive and irritable temperaments, and were more likely to be single and unemployed. Females in the schizophrenia group also tended to score higher in these temperaments compared to their male counterparts. Future studies should consider the possible role of cultural influence on the sampled population. As depressive, cyclothymic and anxious temperaments are significantly present with higher PANSS scores, clinicians and caretakers should take into consideration this dynamic is not a cause and effect but rather a cycle.

## Limitations

While not many studied worked on this topic, final results should be considered preliminary and not enough to generalize. Differences in scores between genders in both the schizophrenia group and healthy controls require a larger sample size. In addition, this was a cross a cross-sectional study and therefore we cannot give evidence of causality. Furthermore, we should be aware of the issue of validity of personality questionnaires, which is not well established in psychotic patients. Finally, results of this study cannot differentiate between depressive temperament and negative symptoms associated with schizophrenia. Future longitudinal studies are recommended in order to study the causality between the latter variables.

## Supplementary Information

**Additional file 1: Figure S1.** Flow diagram. **Table S1.** Comparison of temperaments between patients with schizophrenia and healthy controls. **Table S2.** Comparison of temperaments between patients with schizophrenia and healthy controls by gender.

## Data Availability

All data generated or analyzed during this study are not publicly available to maintain the privacy of the individuals’ identities. The dataset supporting the conclusions is available upon request to the corresponding author.

## Abbreviations

PHC: Psychiatric Hospital of the Cross
TEMPS-A: Affective temperament scale
PANSS: Positive and Negative Syndrome Scale
