# Supplementary material for: Affective temperaments of Lebanese patients with schizophrenia: comparison by gender and severity of psychosis
Source: BMC Res Notes. 2021 Nov 25;14:430. doi: 10.1186/s13104-021-05854-8 (PMC8620946; doi:10.1186/s13104-021-05854-8)
Supplement: Supplementary file 1 — Additional file 1: Figure S1. Flow diagram. Table S1. Comparison of temperaments between patients with schizophrenia and healthy controls. Table S2. Comparison of temperaments between patients with schizophrenia and healthy controls by gender. [file 13104_2021_5854_MOESM1_ESM.docx]

Supplementary Figure 1: Flow diagram

| **Supplementary Table 1: Comparison of temperaments between patients with schizophrenia and healthy controls** | | | |
| --- | --- | --- | --- |
|  | **Schizophrenia patients** | **Healthy controls** | **p-value** |
| **Temperament** |  |  |  |
| Depressive | 0.65 ± 0.15 | 0.37 ± 0.08 | **<0.001** |
| Cyclothymic | 0.51 ± 0.16 | 0.36 ± 0.08 | **<0.001** |
| Hyperthymic | 0.50 ± 0.17 | 0.81 ± 0.06 | **<0.001** |
| Irritable | 0.40 ± 0.21 | 0.31 ± 0.15 | **<0.001** |
| Anxious | 0.53 ± 0.22 | 0.37 ± 0.16 | **<0.001** |

Numbers in bold refer to significant p-value

| **Supplementary Table 2: Comparison of temperaments between patients with schizophrenia and healthy controls by gender.** | | | | | | |
| --- | --- | --- | --- | --- | --- | --- |
|  | **Schizophrenia patients** | | **p-value** | **Healthy controls** | | p-value |
|  | **Male** | **Female** |  | **Male** | **Female** |  |
|  | **Mean ± SD** | **Mean ± SD** |  | **Mean ± SD** | **Mean ± SD** |  |
| **Temperament** |  | |  |  | |  |
| Depressive | 0.59±0.14 | 0.70±0.13 | **<0.001** | 0.34±0.09 | 0.40±0.02 | **<0.001** |
| Cyclothymic | 0.45±0.15 | 0.58±0.15 | **<0.001** | 0.36±0.10 | 0.35±0.02 | **<0.001** |
| Hyperthymic | 0.49±0.16 | 0.50±0.18 | 0.320 | 0.83±0.06 | 0.77±0.03 | **<0.001** |
| Irritable | 0.38±0.21 | 0.41±0.19 | 0.345 | 0.38±0.13 | 0.22±0.10 | **<0.001** |
| Anxious | 0.46±0.21 | 0.61±0.20 | **<0.001** | 0.32±0.17 | 0.43±0.12 | **<0.001** |
